# Macrophage migration inhibitory factor facilitates the therapeutic efficacy of mesenchymal stem cells derived exosomes in acute myocardial infarction through upregulating miR-133a-3p

**DOI:** 10.1186/s12951-021-00808-5

**Published:** 2021-02-27

**Authors:** Wenwu Zhu, Ling Sun, Pengcheng Zhao, Yaowu Liu, Jian Zhang, Yuelin Zhang, Yimei Hong, Yeqian Zhu, Yao Lu, Wei Zhao, Xinguang Chen, Fengxiang Zhang

**Affiliations:** 1 Section of Pacing and Electrophysiology, Division of Cardiology, The First Affiliated Hospital With Nanjing Medical University, Nanjing, Guangzhou Road 300, Nanjing, 210029 People’s Republic of China; 2 Department of Cardiology, The Affiliated Changzhou No. 2 People’s Hospital of Nanjing Medical University, Changzhou, China; 3 Department of Cardiology, Zhongda Hospital of Southeast University, Nanjing, China; 4 Department of Emergency and Critical Care Medicine, Guangdong Provincial People’s Hospital, Guangdong Academy of Medical Sciences, Guangzhou, China

**Keywords:** Exosomes, Macrophage migration inhibitory factor, UcMSCs, MiR-133a-3p, Myocardial infarction

## Abstract

**Background:**

Exosome transplantation is a promising cell-free therapeutic approach for the treatment of ischemic heart disease. The purpose of this study was to explore whether exosomes derived from Macrophage migration inhibitory factor (MIF) engineered umbilical cord MSCs (ucMSCs) exhibit superior cardioprotective effects in a rat model of AMI and reveal the mechanisms underlying it.

**Results:**

Exosomes isolated from ucMSCs (MSC-Exo), MIF engineered ucMSCs (MIF-Exo) and MIF downregulated ucMSCs (siMIF-Exo) were used to investigate cellular protective function in human umbilical vein endothelial cells (HUVECs) and H9C2 cardiomyocytes under hypoxia and serum deprivation (H/SD) and infarcted hearts in rats. Compared with MSC-Exo and siMIF-Exo, MIF-Exo significantly enhanced proliferation, migration, and angiogenesis of HUVECs and inhibited H9C2 cardiomyocyte apoptosis under H/SD in vitro. MIF-Exo also significantly inhibited cardiomyocyte apoptosis, reduced fibrotic area, and improved cardiac function as measured by echocardiography in infarcted rats in vivo. Exosomal miRNAs sequencing and qRT-PCR confirmed miRNA-133a-3p significantly increased in MIF-Exo. The biological effects of HUVECs and H9C2 cardiomyocytes were attenuated with incubation of MIF-Exo and miR-133a-3p inhibitors. These effects were accentuated with incubation of siMIF-Exo and miR-133a-3p mimics that increased the phosphorylation of AKT protein in these cells.

**Conclusion:**

MIF-Exo can provide cardioprotective effects by promoting angiogenesis, inhibiting apoptosis, reducing fibrosis, and preserving heart function in vitro and in vivo. The mechanism in the biological activities of MIF-Exo involves miR-133a-3p and the downstream AKT signaling pathway.

**Supplementary Information:**

The online version contains supplementary material available at 10.1186/s12951-021-00808-5.

## Introduction

Exosomes are small extracellular vesicles with a diameter of 30–150 nm and contain multifarious proteins, mRNAs, microRNAs and additional macromolecules [1]. A growing number of research studies have shown the regulatory role of exosomes in biological processes, including organ crosstalk and intercellular signaling [2–4]. They have anti-apoptotic effects and are used to treat animals in acute myocardial infarction (AMI) [2]. Compared with mesenchymal stem cells (MSCs), exosomes retain the function of parent cells and have many advantages such as long term stability, easy internalization into receptor cells, minimal immunological rejection, and little potential for tumorigenesis [5]. Thus, exosome transplantation is a promising cell-free therapeutic approach for the treatment of ischemic heart disease.

Included in the cargo of exosomes, microRNAs (miRNAs) have been demonstrated to control important processes that conduce to the pathological consequences of AMI [6]. miRNAs are small non-coding RNAs, regulating gene expression, leading to degradation or translation inhibition of the mRNAs [7]. A large number of studies have shown that miRNAs are functional in apoptosis, angiogenesis, and fibrosis after myocardial infarction [8, 9].

Exosomes are secreted from many kinds of cell types including MSCs and macrophage migration inhibitory factor (MIF) engineered MSCs [10]. MIF is a pro-inflammatory cytokine that is widely expressed in many kinds of cells including MSCs [11]. Recent studies have shown that MIF plays an important role in cell survival and proliferation, and our previous study demonstrated that exosomes from MSCs with overexpression of MIF had better cardiac protection in rats with AMI [12, 13]. In the present study, umbilical cord MSCs (ucMSCs) were infected with lentivirus containing MIF (MIF-MSC). Exosomes from MIF-MSC were then isolated and the mechanism behind this cardiac protective function of exosomes from MIF engineered ucMSCs was investigated. In vitro*,* results showed that exosomes from MIF engineered ucMSCs (MIF-Exo) significantly improved cell survival, blood vessel formation, and cell migration over control non-engineered ucMSCs (MSC-Exo). In vivo, results demonstrated that MIF-Exo reduced fibrosis area, promoted capillary formation, inhibited apoptosis, and improved overall heart function better than MSC-Exo. miRNA sequencing and quantitative real-time PCR (qRT-PCR) showed that miR-133a-3p expression significantly increased in MIF-Exo (vs. MSC-Exo). These cardioprotective effects were attenuated with inhibition of exosomal miR-133a-3p, indicating a potential mediation role of MIF-Exo in the ischemic heart.

## Results

### Characterization of uMSCs and exosomes derived from ucMSCs

**Fig. 1:**
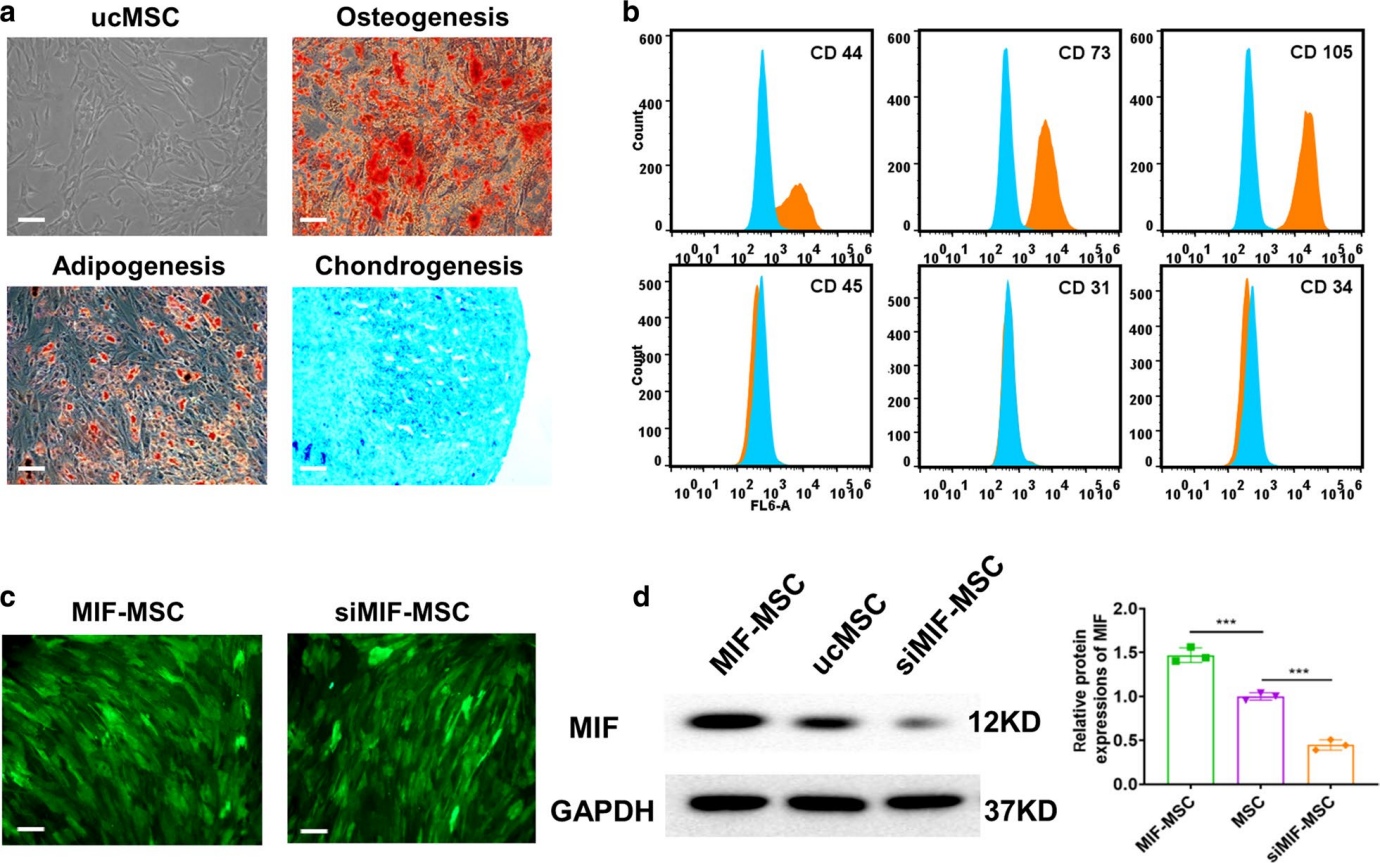
Identification of human umbilical cord mesenchymal stem cell. **a** Morphology and multiple differentiation potential of ucMSCs. Scale bar = 100 μm. **b** Surface markers profiling of ucMSCs. **c** Successful lentiviral transfection was confirmed by positive green fluorescence under microscope in both MIF-MSC and siMIF-MSC groups. **d** Western blot images showed MIF protein levels in MIF-MSC, MSC and siMIF-MSC groups. ***P < 0.001, MIF-MSC vs. ucMSCs; ***P < 0.001, ucMSCs vs. siMIF-MSC

Multiple differentiation potential of ucMSCs towards osteogenesis, adipogenesis, and chondrogenesis of ucMSCs were confirmed by Alcian blue staining, Oil red staining and Alizarin red staining (Fig. 1a). ucMSCs were positive for surface markers of CD44, CD73, CD105, and negative for markers of CD45, CD31 and CD34 (Fig. 1b). To verify whether lentiviral modification was conducted successfully, green fluorescence was observed in both MIF-engineered ucMSCs and MIF downregulated ucMSCs (siMIF-MSC) by fluorescent microscopy (Fig. 1c). In addition, western blot showed that MIF protein levels significantly decreased in siMIF-MSC compared with ucMSCs and MIF-MSC (Fig. 1d).

**Fig. 2:**
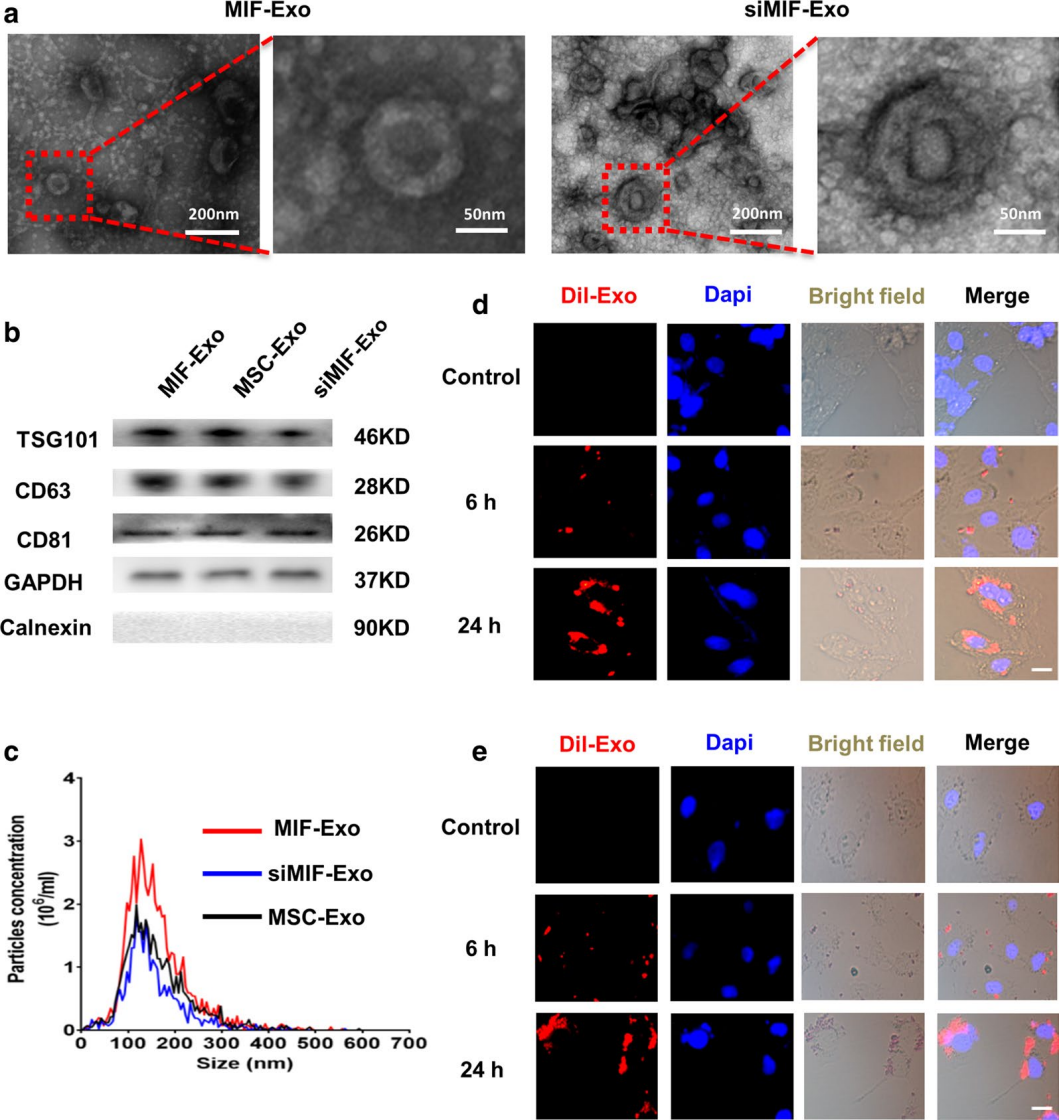
Characterization of exosomes derived from ucMSCs. **a** Cup-shaped morphology of purified MIF-Exo and siMIF-Exo (arrowhead) assessed by TEM. **b** Representative images of western blot showing the exosomal protein markers in MIF-MSC, ucMSCs and siMIF-MSC groups. **c** The particle size distribution and particle concentration were analyzed by nanoparticle tracking analysis (n = 3 biological replicates for each group). Confocal images showed that red fluorescence of dye Dil labeled exosomes were endocytosed by H9C2 (**d**) and HUVECs (**e**) 6 and 24 h after incubation. Scale bar = 20 μm

Exosomes were then isolated from MIF-MSC and siMIF-MSC. TEM showed that the morphology of exosomes from MIF-Exo and siMIF-Exo was a typical cup-shaped structure, with a size of around 100 nm (Fig. 2a). Exosome specific markers of TSG101, CD81 and CD63 were positive in MIF-Exo, MSC-Exo, and siMIF-Exo. GAPDH was used as a loading control and Calnexin were not detected among exosome proteins (Fig. 2b). The particle size and concentration of Exo were similar among the three groups (Fig. 2c). After labelling exosomes with Dil (10 μmol/L, 1μL) for 6 h, they were then co-cultured with H9C2 cells and HUVECs for 6 h and 24 h under H/SD. Confocal images showed that both H9C2 and HUVECs could uptake labeled exosomes and exosome absorption by cells was presented in a time dependent manner (Fig. 2d, e).

### MIF-Exo conferred better protective effects on HUVECs and H9C2 cardiomyocytes than MSC-Exo under hypoxia and serum deprivation (H/SD) in vitro

In order to examine whether MIF-Exo have superior cellular protective effects when compared with MSC-Exo and siMIF-Exo, the effects of these preparations on neovascularization, migration, proliferation, and apoptosis in HUVECs and H9C2 cells were evaluated.

**Fig. 3:**
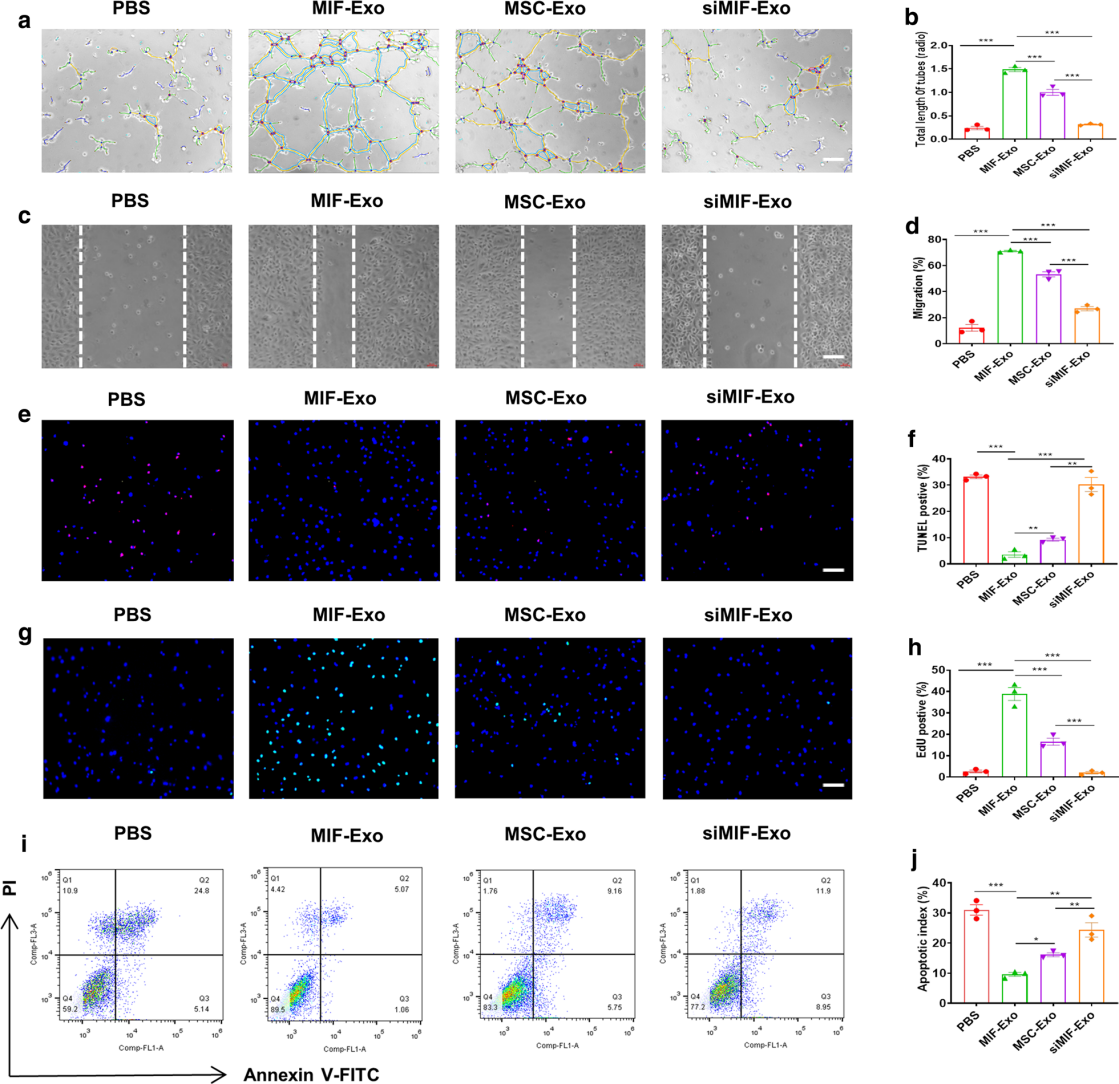
MIF-Exo exhibited more significant protective effects on HUVECs and H9C2 cardiomycytes than MSC-Exo in vitro. Tube formation of HUVECs incubated with PBS, MIF-Exo, MSC-Exo and siMIF-Exo (**a**), and quantification analysis (**b**). Scale bar = 100 μm. (n = 3 biological replicates for each group). Migration was monitored for 12 h after scratching in HUVECs cultured with PBS, MIF-Exo, MSC-Exo and siMIF-Exo (**c**), and quantification analysis (**d**). Scale bar = 100 μm (n = 3 biological replicates for each group). In H/SD, DAPI nucleic acid stained for apoptosis of HUVECs after culturing with PBS, MIF-Exo, MSC-Exo and siMIF-Exo. Red point indicated apoptotic cells (**e**), and quantification analysis (**f**). Scale bar = 100 μm. (n = 3 biological replicates for each group; 5 random fields for each biological replicate). EdU positive cells were in PBS, MIF-Exo, MSC-Exo and siMIF-Exo (**g**), and quantification analysis (**h**). Scale bar = 100 μm. (n = 3 biological replicates for each group; 5 random fields for each biological replicate). In H/SD, apoptosis of H9C2 after incubating with PBS, MIF-Exo, MSC-Exo and siMIF-Exo (**i**), and quantification analysis (**j**). Continuous variables and categorical variables were described by means ± SEM and percentages. (n = 3 biological replicates for each group). *P < 0.05; **P < 0.01; ***P < 0.001

Angiogenesis of HUVECs significantly increased in the MIF-Exo group compared with PBS, MSC-Exo, and siMIF-Exo groups, while there was no significant difference between the siMIF-Exo group and PBS group (Fig. 3a, b). Migration rate of HUVECs was also significantly enhanced in the MIF-Exo group when compared with PBS, MSC-Exo, and siMIF-Exo groups (Fig. 3c, d). TUNEL and flow cytometry both showed that the percentage of apoptosis cells was significantly reduced in the MIF-Exo group as compared with PBS, MSC-Exo, and siMIF-Exo groups, while there was no significant difference between siMIF-Exo group and PBS group (Fig. 3e-j). EdU positive cells significantly increased in the MIF-Exo group when compared with PBS, MSC-Exo and siMIF-Exo groups (Fig. 3g, h). These results suggest that MIF-Exo confer superior cellular protective effects on HUVECs and H9C2 cardiomyocytes compared to MSC-Exo under H/SD in vitro.

### *MIF-Exo effectively preserved cardiac function in rats with MI *in vivo

**Fig. 4:**
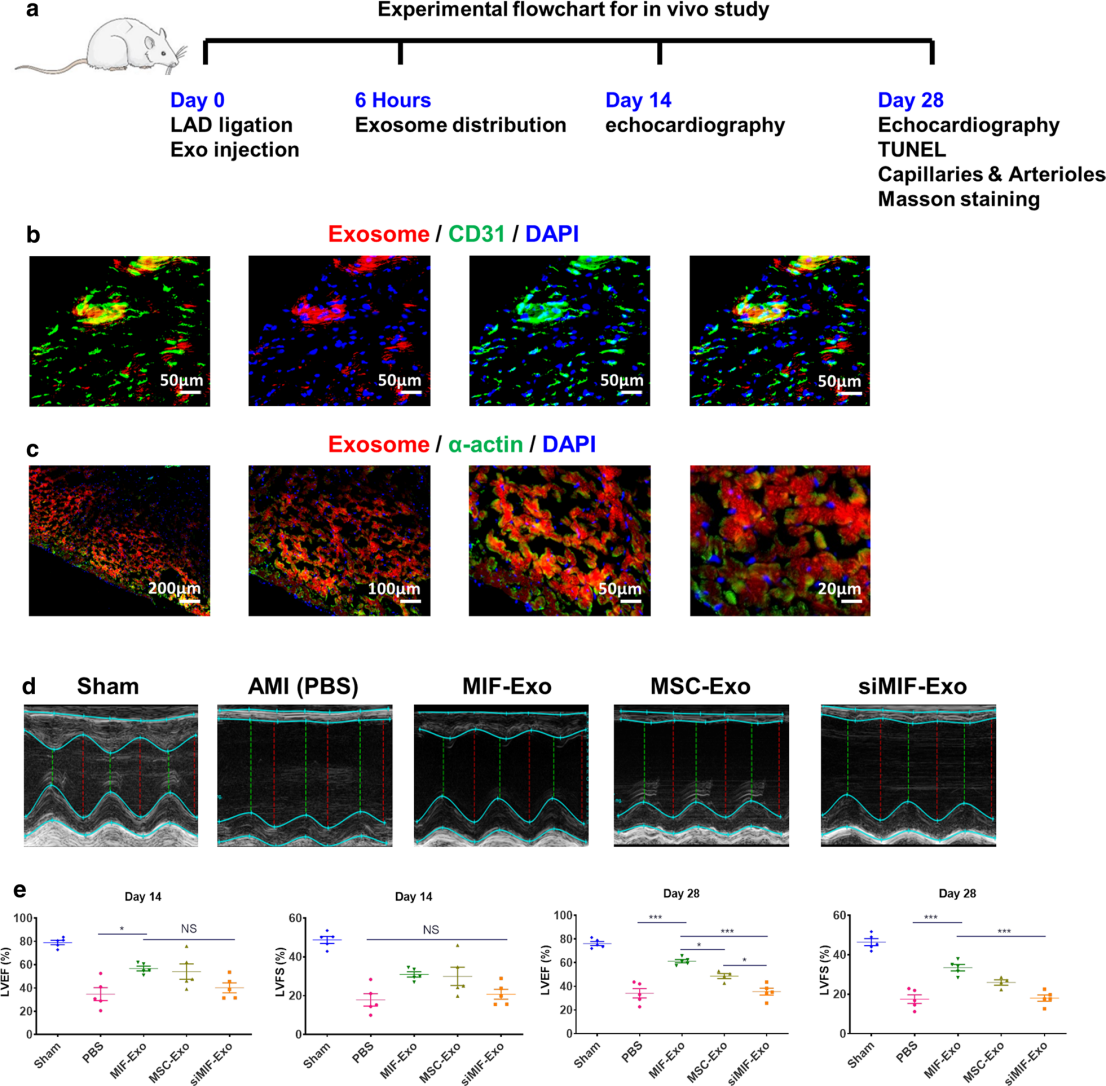
MIF-Exo effectively preserved cardiac function in rats with MI in vivo. **a** The flowchart of experimental design in vivo. The protein expression level of CD31 (**b**) and a-actin (**c**) was detected by immunofluorescence after Dil labeled exosomes taken by endothelial cells and cardiomyocytes 6 h after intramyocardial injection. (n = 3 biological replicates; 4 random fields for each animal). LVEF and LVFS were measured 2 and 4 weeks post MI (**d**), and quantification analysis (**f**). (2 weeks: n = 5 animals for each group; 4 weeks: n = 4 animals for MSC-Exo group, n = 5 animals for the other groups). Continuous variables and categorical variables were described by means ± SEM and percentages. *P < 0.05; ***P < 0.001; NS, not significant

In order to evaluate the benefits of MIF-Exo cardiac function in vivo, PBS, MIF-Exo, MSC-Exo, and siMIF-Exo were injected at the border of infarction area in rats (Fig. 4a). Six hours after establishing the MI model and exosome injection, large areas were positive for Dil labeled exosomes in the myocardium (Fig. 4b) and near the endothelium (Fig. 4c). Two weeks after MI, LVEF was significantly increased in MIF-Exo group compared with PBS group. Four weeks after MI, LVEF significantly improved and LVFS significantly increased in the MIF-Exo group compared with PBS, MSC-Exo and siMIF-Exo groups (Fig. 4d, e). These results suggested that MIF-Exo play a potential role in preserving heart function in infarcted rats.

### *MIF-Exo promote angiogenesis and cardiomyocyte survival in rats with MI *in vivo

**Fig. 5:**
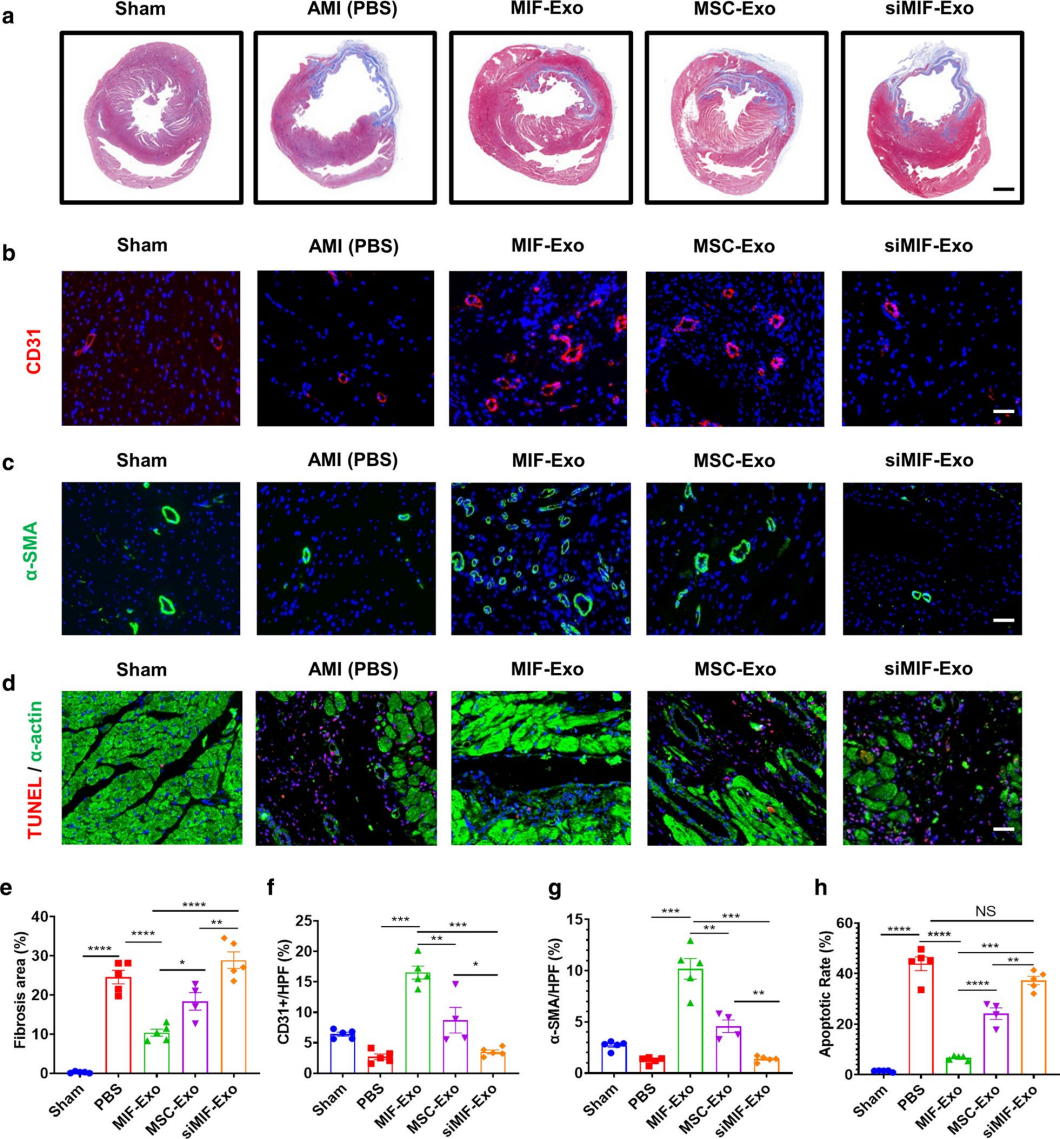
MIF-Exo promoted angiogenesis and cardiomyocyte survival in infarcted hearts. Fibrosis area was in Sham, PBS, MIF-Exo, MSC-Exo and siMIF-Exo groups (**a**), and quantification analysis (**e**). Scale bar = 2 mm. CD31 positively stained capillaries at the border zone 4 weeks post MI. Left ventricle was selected in Sham group (**b**), and quantification analysis (**f**). Scale bar = 50 μm. α-SMA positively stained arterioles in the infarct area week 4 after MI. Left ventricle was selected (**c**), and quantification analysis (**g**). Scale bar = 50 μm. TUNEL staining at the border zone 4 weeks after MI (**d**), and quantification analysis (**h**). Scale bar = 50 μm. (n = 4 animals for MSC-Exo group, n = 5 animals for the other groups, 4 random fields per animal). Continuous variables and categorical variables were described by means ± SEM and percentages. *P < 0.05; **P < 0.01; ***P < 0.001; ****P < 0.0001; NS, not significant

The role of MIF-Exo was also investigated to determine any therapeutic potential on fibrosis, angiogenesis, and anti-apoptosis in vivo. Masson staining indicated that the fibrosis area significantly decreased in the MIF-Exo group compared with PBS, MSC-Ex, and siMIF-Exo groups (Fig. 5a, e). To reveal the mechanism of this exosomal treatment, immunofluorescence with antibodies against CD31 and α-SMA were used to stain capillaries and arteriole. Four weeks after MI, the capillary density significantly increased in the MIF-Exo group compared with PBS, MSC-Exo, and siMIF-Exo groups (Fig. 5b, f). The arteriolar density was also increased in the MIF-Exo group compared with the other three groups (Fig. 5c, g). TUNEL showed that apoptotic cells significantly decreased in the MIF-Exo group compared with PBS, MSC-Exo, and siMIF-Exo groups (Fig. 5d, h). Thus, by reducing fibrosis area, promoting angiogenesis, and protecting cells from apoptosis, MIF-Exo confer significant benefits for improving cardiac repair.

### miR-133a-3p expression significantly increased in MIF-Exo

**Fig. 6:**
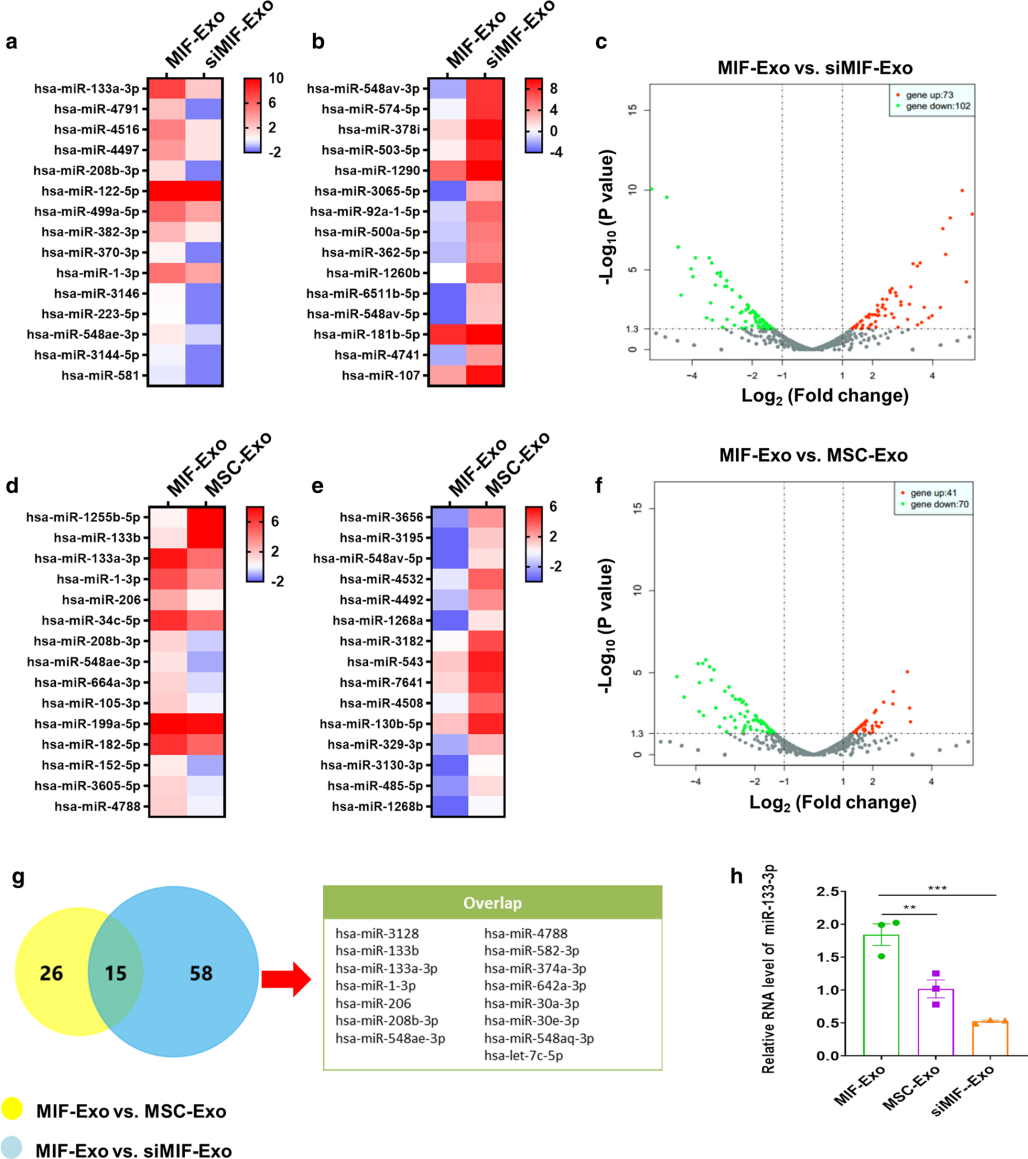
miR-133a-3p level increased in MIF-Exo. Heat map based on exosomal miRNAs sequence values (red represents high expression and blue represents low expression) between MIF-Exo and siMIF-Exo. Top 15 upregulated miRNAs (**a**) and top 15 downregulated miRNAs (b**B**) were shown in MIF-Exo group. **c** Volcano plot showed log_2_ (Fold change) on x-axis and -log_10_ (P value) on y-axis. Heat map based on exosomal miRNAs sequence values between MIF-Exo and MSC-Exo group. Top 15 upregulated miRNAs (**d**) and top 15 downregulated miRNAs (**e**) in MIF-Exo group are shown. **f** Volcano plot showed log_2_ (Fold change) on the x-axis and -log_10_ (P value) on the y-axis. **g** In these upregulated miRNAs, 15 miRNAs were overlapped. **h** miR-133a-3p levels in MIF-Exo, MSC-Exo and siMIF-Exo groups were measured by qRT-PCR. (n = 3 biological replicates, 3 technical replicates for each biological replicate). All data were mean ± SEM. **P < 0.01; ***P < 0.001; NS, not significant

In order to explore the molecular mechanisms involved in the cardioprotective effects of MIF-Exo, exosomal miRNAs sequencing was performed on MIF-Exo, MSC-Exo, and siMIF-Exo. There were 73 upregulated and 102 downregulated miRNAs in MIF-Exo compared with siMIF-Exo (Fig. 6a, c). 41 upregulated and 70 downregulated miRNAs were in MIF-Exo compared with MSC-Exo (Fig. 6d, f). The top 15 upregulated and downregulated miRNAs were listed (Fig. 6a, b, d, e). In these upregulated miRNAs, there were 15 miRNAs that overlapped (Fig. 6g). Of them, qRT-PCR confirmed that miRNA-133a-3p most significantly increased in MIF-Exo compared with MSC-Exo and siMIF-Exo (Fig. 6h). It has also been previously reported that miRNA-133a-3p is associated with cardiovascular disease regulation [14, 15] and thus became the focus of our study.

### *Gain- and loss- of function of exosomal miR-133a-3p on angiogenesis, proliferation, and apoptosis in HUVECs and H9C2 cells *in vitro

**Fig. 7:**
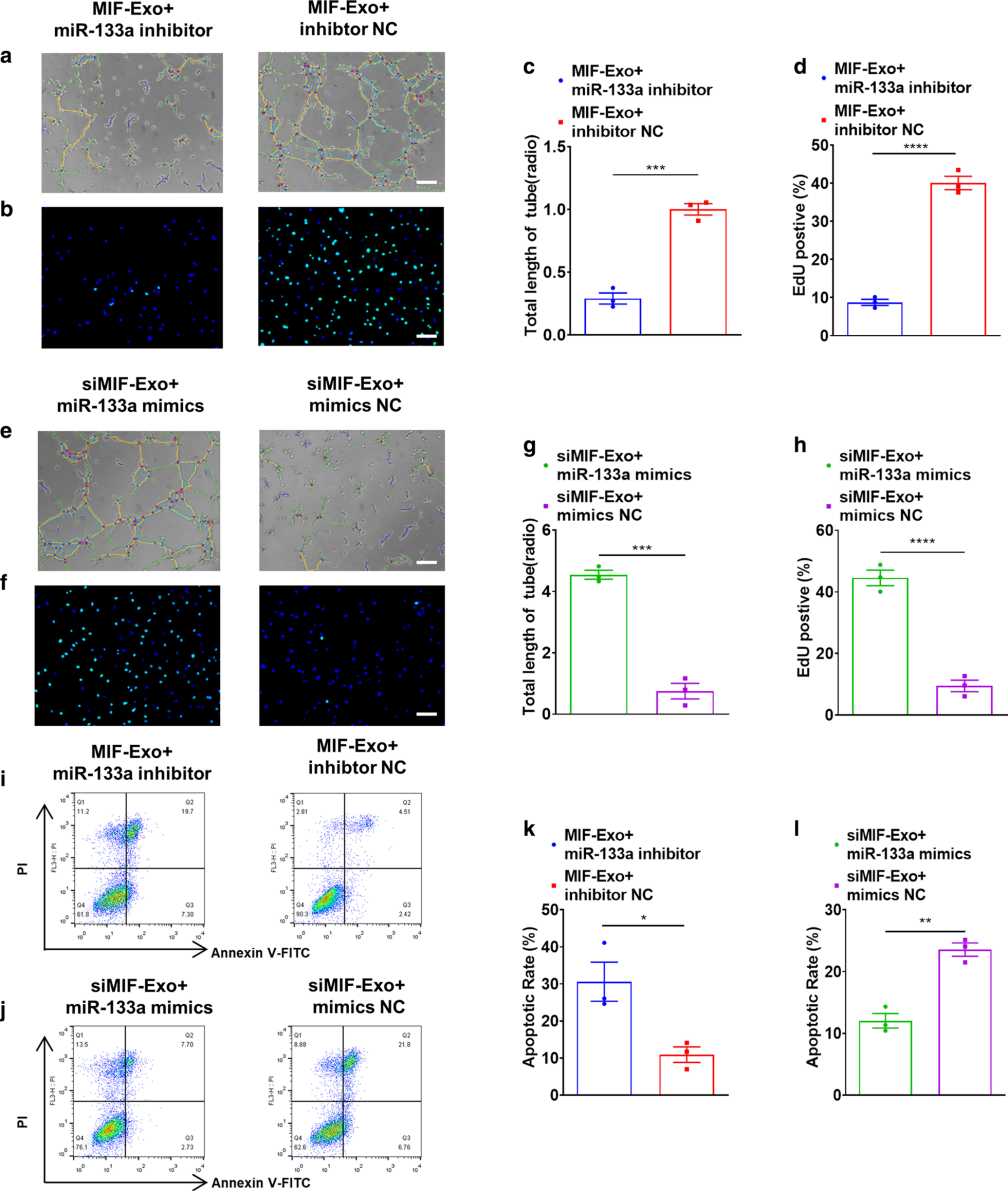
Gain and loss function of exosomal miR-133a-3p on pro-angiogenisis, proliferation, and apoptosis in HUVECs and H9c2 cells in vitro*.* Proangiogenic effects of HUVECs were diminished with incubation of MIF-Exo and miR-133a-3p inhibitor (**a**), and quantification analysis (**c**). Scale bar: 100 μm. (n = 3 biological replicates for each group). Proliferation effects of HUVECs were attenuated with incubation of MIF-Exo and miR-133a-3p inhibitor (**b**), and quantification analysis (**d**). Scale bar: 100 μm. (n = 3 biological replicates for each group; 5 random fields for each biological replicate). Proangiogenic activity of HUVECs restored with incubation of siMIF-Exo and miR-133a-3p mimics (**e**), and quantification analysis (**g**). Scale bar: 100 μm. (n = 3 biological replicates for each group). Proliferation of HUVECs rescued with incubation of siMIF-Exo and miR-133a-3p mimics (**f**), and quantification analysis (**h**). Scale bar: 100 μm. (n = 3 biological replicates; 5 random fields for each biological replicate). Anti-apoptotic ability of H9c2 cells reduced with incubation of MIF-Exo and miR-133a-3p inhibitor (**i**), and quantification analysis (**k**). Scale bar: 100 μm C. (n = 3 biological replicates for each group). Anti-apoptotic ability of H9c2 cells rescued with incubation of siMIF-Exo and miR-133a-3p mimics (**j**), and quantification analysis (**l**). (n = 3 biological replicates for each group). Continuous variables and categorical variables were described by means ± SEM and percentages. *P < 0.05; **P < 0.01; ***P < 0.001; ****P < 0.0001

Gain- and loss- of function studies were carried out to identify the effects of exosomal miR-133a-3p on angiogenesis, proliferation, and apoptosis in HUVECs and H9C2 cells in vitro. HUVECs and H9C2 cells were transfected by miR-133a-3p mimics, miR-133a-3p inhibitor, and their negative controls (NC) successfully and were validated by qRT-PCR (Additional file 1: Figure S1). We found that incubation of MIF-Exo and miR-133a inhibitor could significantly attenuate the effects of MIF-Exo on angiogenesis (Fig. 7a, c), and proliferation (Fig. 7b, d) in HUVECs compared with MIF-Exo and miR-133a inhibitor NC. Incubation of siMIF-Exo and miR-133a mimics also accentuated the effects of MIF-Exo on promoting tube formation (Fig. 7e, g) and proliferation (Fig. 7f, h) in HUVECs compared with siMIF-Exo and miR-133a mimics NC. We also found that co-culturing of MIF-Exo and miR-133a inhibitor could increase apoptotic cell number (Fig. 7i, k) in H9C2 cells when compared with MIF-Exo and miR-133a inhibitor NC. And co-culturing of siMIF-Exo and miR-133a mimics could suppress pro-apoptotic effects compared with siMIF-Exo and miR-133a mimics NC (Fig. 7j, l). We also found that incubation of MIF-Exo and miR-133a inhibitor could significantly attenuate the effects of MIF-Exo on pro-angiogenesis and proliferation in HUVECs compared with MIF-Exo group. Moreover, we found that co-culturing of MIF-Exo and miR-133a inhibitor could increase apoptotic cell number in H9C2 cells (Additional file 1: Figure S2) when compared with MIF-Exo group. These results indicate that miR-133a-3p plays a key role in MIF-Exo induced cellular protection.

### *miR-133a-3p inhibited apoptosis, enhanced angiogenesis, and promoted proliferation *via* AKT pathway in HUVECs and H9C2 cells, and promoted VEGF protein expression in HUVECs*

**Fig. 8:**
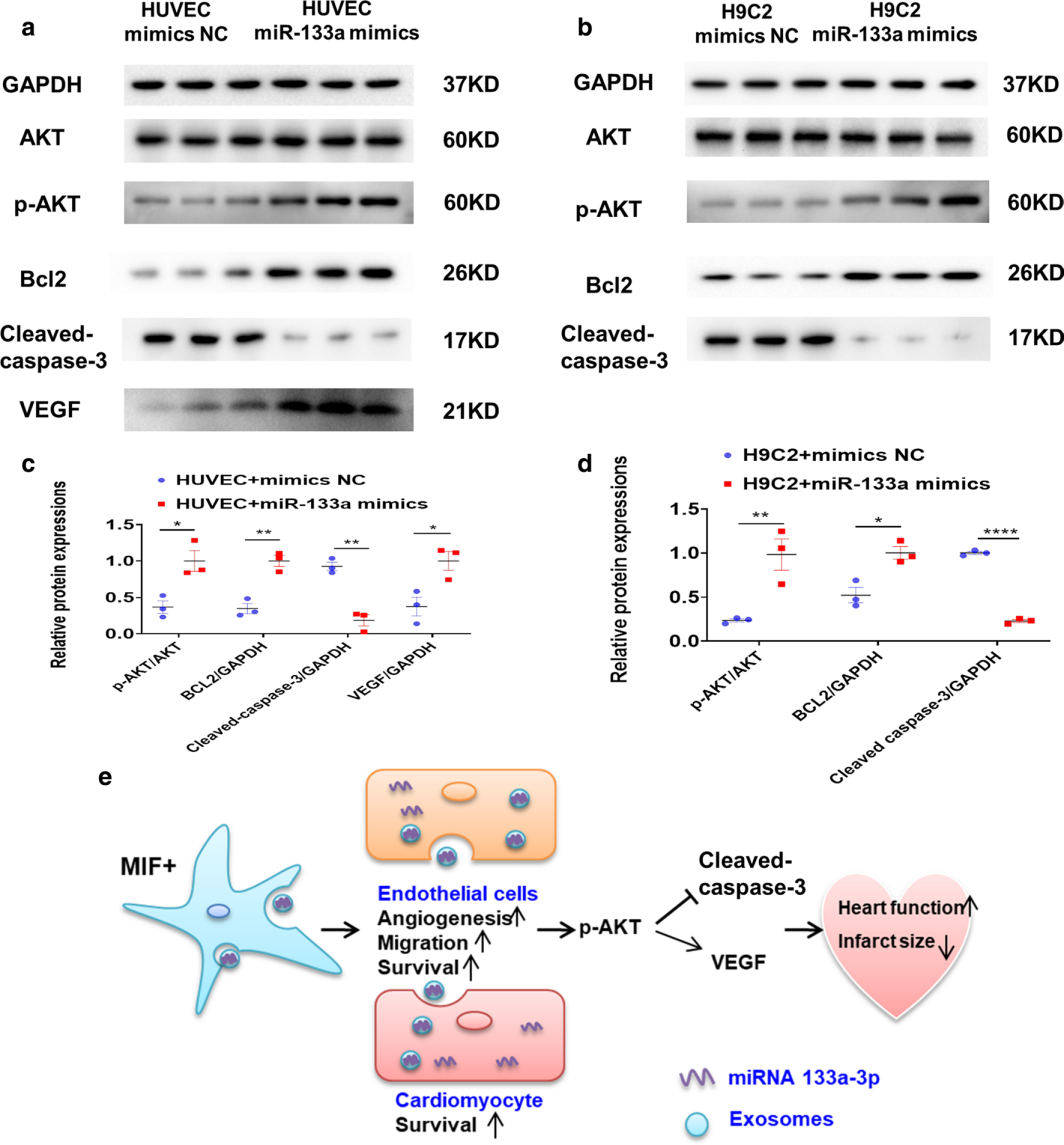
Exosomal miR-133a-3p inhibited cardiomyocyte apoptosis, promoted angiogenesis by AKT signal pathway, and improved the protein expression of VEGF in HUVECs. The protein levels of p-AKT, AKT, Bcl-2, cleaved caspase-3 and VEGF in HUVECs (**a**), and quantification analysis (**c**). (n = 3 biological replicates). The protein levels of p-AKT, AKT, Bcl-2, and cleaved caspase-3 in H9C2 cells (**b**), and quantification analysis (**d**). (n = 3 biological replicates). Continuous variables and categorical variables were described by means ± SEM and percentages. *P < 0.05; **P < 0.01; ****P < 0.0001. **e** Schematic showed the working model of this study

To further identify the potential signal pathway involved with miR-133a-3p biological activity, miR-133a-3p mimics and miR-133a-3p mimics NC were incubated with H9C2 cells and HUVECs. In HUVECs, AKT protein phosphorylation increased, followed by Bcl-2 protein level upregulated and cleaved caspase-3 downregulated; the protein expression of VEGF also increased, in miR-133a-3p mimics group. These effects were reversed with mimics NC (Fig. 8a, c). In H9C2 cells, an enhanced AKT phosphorylation in miR-133a-3p mimics group, and adjusting its downstream targets Bcl-2 (upregulated) and cleaved caspase-3 (downregulated) (Fig. 8b, d). In summary, these data suggest that miR-133a-3p inhibits cardiomyocyte apoptosis, enhances angiogenesis, and increases proliferation via AKT signal pathway, and increases VEGF levels in HUVECs.

## Discussion

The results of this study demonstrate that MIF-Exo promotes migration, inhibits apoptosis, and improves tube like structure formation under H/SD in HUVECs or H9C2 cardiomyocytes. Moreover, findings demonstrated that MIF-Exo was superior in preserving cardiac function and inhibiting fibrosis compared with MSC-Exo. Our findings also reveal a novel mechanism by which the protective effects of MIF-Exo are at least partially mediated by miR-133a-3p through AKT signal pathway (Fig. 8e).

Genetically modified stem cells have been effectively applied to repair injured hearts. A recent study showed that MSCs overexpressing IL-33 enhanced heart function and reduced inflammation in rats with MI [16]. Another study showed that aged MSCs overexpressing ERBB4 reduced myocardial infarct size and improved cardiac function [17]. Yu et al. also reported that exosomes from MSCs overexpressing GATA-4 could reduce cardiac injury [18]. Ma et al. reported that exosomes from MSCs modified with AKT could promote cardiac angiogenesis and regeneration [19]. Our previous study also showed that MIF rejuvenates aged human MSCs and improves myocardial repair [12]. MSCs achieve cardiac protective function mainly through paracrine factors such as exosomes [20–23]. Recent studies have shown that exosomes derived from MSCs reduce myocardial ischemic injury and preserve cardiac function after MI [24, 25]. Compared with conventional stem cell transplantation therapy, exosome therapy showed no teratoma development, minimum immunogenicity, and little potential for tumorigenesis [26, 27]. It is a promising new cell free therapy for cardiac repair. Compared with liposomes, nanoparticles, microspheres, microemulsions and other synthetic drug loading systems, the endogeneity of exosomes is a natural and unique advantage [28–31]. However, more extensive testing and validation of exosomes therapeutics are needed to assure safety and efficacy [32].

The data in this study supports a model in which exosomes released by MIF-MSC produce anti-apoptotic and pro-angiogenic effects that protect cardiac function through a mechanism involving miR-133a-3p. Exosomes are extracellular vesicles containing miRNAs et al., and they are absorbed by target cells. Exosomes regulate intracellular signal pathways [33]. Recent studies have shown that exosomal miRNAs play an important role in many biological processes [34]. In this current study, expression profiles of miRNAs in MIF-Exo revealed that miR-133a-3p was significantly increased in MIF-Exo and reduced in siMIF-Exo compared with MSC-Exo. This indicates that the expression of miR-133a-3p changed dynamically with overexpression or knockdown of the MIF gene in ucMSCs.

It is reported that miR-133a-3p plays a very important role in heart and muscle development [35–37]. The circulating expression of miR-133a-3p increased significantly in AMI patients compared with normal people [38]. Thus, it could be considered as a diagnostic biomarker for AMI patients [39]. Moreover, it was also reported that miR-133a-3p potentially suppresses apoptosis and regulates fibrosis after MI [14]. In this study, we verified that exosomes from MIF engineered ucMSCs could reduce fibrosis area and improve cardiac function in ischemic hearts. The protective effects of MIF-Exo at least partially mediated by miR-133a-3p.

We found that miR-133a-3p mimics promoted phosphorylation of AKT in HUVECs or H9C2 cells, indicating a possible molecular mechanism for the positive role of miR-133a-3p in cardioprotection. Survival signals AKT pathway has been reported as a key target in cardioprotection [40, 41]. miR-133a has also been well documented as a suppressor of cardiac remodeling and the mechanisms are also involving many target genes and pathways including AKT pathway [42, 43]. These evidences further support the cardioprotective effects of miR-133a-3p via enhancing AKT signal pathway in cardiomyocytes and endothelial cells. However, further studies are still needed to elucidate the downstream mechanisms of cardioprotective effects of exosomal miR-133a-3p in physiology and pathophysiology.

There are some limitations to this study. First, other exosomal miRNAs may also contribute to cardioprotection effects of MIF-Exo. We did not test and validate the expression and effects of those miRNAs. Second, the expression of miR-133a-3p increased significantly in MIF-Exo, but the mechanisms of miR-133a-3p were regulated by overexpression of MIF in ucMSCs have not been clarified. Third, although we confirmed the protective effects of miR-133a-3p enriched exosomes, further studies are needed to verify the safety and efficacy before use can be considered as a potential cell free therapy for cardiac repair in clinical practice.

MIF engineered ucMSCs derived exosomes enhance the effects by promoting angiogenesis, improving proliferation, inhibiting apoptosis, reducing fibrosis, and preserving heart function both in vitro and in vivo. miR-133a-3p and its downstream AKT signal pathway were involved in the biological activities of MIF-Exo.

## Materials and methods

### Ethics statements

All animal care and surgical procedures were performed with approval from the Nanjing Medical University Animal Care and Use Committee (IACUC-1709019).

### Cell culture and identification

Umbilical cord MSCs from healthy donors were purchased from the Clinical Center of Reproductive Medicine in Nanjing. ucMSCs, H9C2 cells, and human umbilical vein endothelial cells (HUVECs) were cultured in Dulbecco’s modified Eagle medium (DMEM, Gibco, USA) supplemented with 10% foetal bovine serum (FBS, Gibco, USA), 100 U/mL penicillin, 100 μg/Ml streptomycin and 110 mg/mL sodium pyruvate. All cells were incubated at 37 ℃ in a humidified atmosphere containing 5% CO2. ucMSCs used for experiments were between passages 3 and 6.

Umbilical cord MSCs were characterized by the expression of cell surface markers. They were washed with 2% FBS/phosphate-buffered saline (PBS) and incubated at 4 ℃ for 30 min with 5 µl of a monoclonal antibody specific for CD31, CD34, CD45, CD73, CD44 and CD105 (1:200, BD Biosciences, San Jose, CA, USA). Unstained ucMSCs were used as controls. FACS Canto II (BD Biosciences, San Jose, CA, USA) was used for cytometry analysis.

### Exosome extraction and identification

Exosomes were isolated as previously reported [10]. ucMSCs were cultured to 80% confluence in the complete medium, washed three times with PBS, and subsequently cultured with exosomes free DMEM for 48 h. The conditioned medium was collected and centrifuged at 1500*g* for 30 min to remove apoptotic bodies and cell debris followed incubation with RiboTM Exosome Isolation Reagent (RiboBio, China) for 12 h at 4 ℃. The supernatant was centrifuged at 2,000 g for 30 min. The supernatant was discarded, and the pellet was suspended in PBS and stored at − 80 ℃. BCA kit (Thermo, USA) was used to analyze concentration. Western blotting was used to identify surface markers of exosomes including TSG101 (1:1000, 14,497, (Proteintech, USA), CD63 (1:1000, 25,682, (Proteintech, USA) and CD81 (1:1000, 66,866, (Proteintech, USA). Transmission electron microscopy (TEM, HITACHI, H-600IV, Japan) Nanoparticle Tracking Analysis (NTA, Malvern Instruments, UK) were used to determine particle morphology and the particle size distributions of isolated exosomes.

To evaluate whether exosomes could be absorbed by cells, 1 μmol Dil (a red fluorescent cell linker for general cell membrane labeling) was used for labeling exosomes, and then Dil labeled exosomes were incubated with target cells for 6 h and 24 h. Nuclei were stained with 4′, 6-diamidino-2-phenylindole (DAPI). Confocal images were taken by Zeiss laser-scanning confocal microscope (LSM5 Live, Carl Zeiss, Germany).

### RNA extraction and quantitative real-time PCR (qRT-PCR)

RNA was extracted from exosomes and cells by TRIzol (Invitrogen, Carlsbad, CA, USA) according to the protocols of manufacturer. Spectrophotometer (NanoDrop‐2000, ThermoFisher Scientific) was to inspect the quantity and quality of RNA. Then miRNAs were reverse transcribed by miRNAs reverse transcription kit (Applied Biosystems) by using thermal circulatory apparatus (Applied Biosystems, Foster City, CA, USA). qRT‐PCR was conducted using a SYBR® Green PCR Master Mix (Applied Biosystems) following the instructions. PCR cycling conditions were 95 °C for 5 min, 40 cycles of 95 °C for 10 s, and 60 °C for 30 s. Gene expression data were standardized with the values for Cel-miR-39 (exosomal) and U6 (cellular). The sequences of primers used in the study were shown in Additional file 1: Table S1. All samples were measured in triplicate.

### Western blotting

Cells were lysed in a lysis buffer (Cell Signaling Technology, USA) supplemented with protease inhibitors (Calbiochem, USA) at 4 °C for 30 min, while the exosomes were lysed in 20 μL lysis buffer at 4 °C for 10 min. Total protein concentration was quantified using the BCA protein assay kit (Pierce, USA). Western blotting was performed according to the standard protocol as previously described [12]. Antibodies used were as follows: phosphorylated-AKT (p-AKT) (1:1000, 4060, Cell Signaling Technology), AKT (1:1000, 4691, Cell Signaling Technology); Cleaved caspase-3 (1:1000, 29,034, Signalway Antibody), Bcl-2 (1:1000, ab196495, Abcam), vascular endothelial growth factor (1:1000, VEGF, ab52917, Abcam), glyceraldehyde-3-phosphate dehydrogenase (GAPDH) (1:1000, 5174, Cell Signaling Technology), Calnexin (1:1000, 2679, Cell Signaling Technology) and horseradish peroxidase-conjugated secondary antibody (1:5000, Biosharp). The bands were visualized by using enhanced chemiluminescence reagents and analyzed with a gel documentation system (iBrightCL1000, Invitrogen and Image Lab Software version 3.0).

### Exosomal miRNA sequencing

The miRNAs sequencing was carried out in MIF-Exo, MSC-Exo, and siMIF-Exo. Exosomal miRNA-seq analysis was performed by RiboBio (Guangzhou, China) using the Illumina HiSeq 2500 instrument. Differentially expressed miRNAs were identified through |log2(fold change)|≥ 1 and P-value < 0.05 with the threshold set for up- and downregulated genes. Bioinformatics analyses including differentially expressed miRNA analysis, prediction of target genes of miRNA, gene ontology (GO) analysis, and Kyoto Encyclopedia of Genes and Genomes (KEGG) pathway enrichment analysis were also performed by RiboBio.

### miRNAs transfection

Transfection of miR-133-3p mimics (50 nmol/L), miR-133-3p inhibitors (100 nmol/L) and their negative controls (50–100 nmol/L) were carried out using Lipofectamine 2000 (Invitrogen, USA) according to the instructions of manufacturer. Briefly, cardiac cells were cultured to 70% confluence. miR-133a-3p mimics, miR-133a-3p inhibitor and their negative controls were mixed with a transfection reagent, then added to the cell culture at a final concentration of 50–100 nmol/L. Transfection efficiency was determined by performing qRT-PCR. The sequences of miRNAs transfected were shown in Additional file 1: Table S2.

### Lentiviral constructions and infection

The lentivirus construction in this study was obtained from GENECHEM (Shanghai, China). Two lentiviruses recombinant vectors were constructed. One is pLenti-EF1a-P2A-Puro-NRCMV-MIF-3Flag, used as MIF (overexpression of MIF) virus; and the other, hU6-MCS-Ubiquitin-EGFP-IRES-puromycin, served as siMIF (knocking down of MIF) virus. The sequences of MIF and siMIF used in the study were shown in Additional file 1: Table S3. ucMSCs were seeded in 24 wells plates, when reached 50% confluence, they infected with MIF overexpression lentivirus or MIF knockdown lentivirus. Pools of stable transductions were generated by selection using puromycin (0.75 μg/ml) for three days. Fluorescent signals were viewed under the microscope. MIF expression was evaluated by Western blotting.

### Co-culture experiment

HUVECs or H9C2 cardiomyocytes were seeded in 6 wells or 24 wells plates, once reached 70%–80% confluence, cells were incubated with PBS, MIF-Exo, MSC-Exo, and siMIF-Exo (100 μg/mL) for 24 h. After incubation, the cells were subjected to hypoxia and serum deprivation (H/SD) for another 12 h, and they were evaluated by further experiments (tube formation assay, edU and migration assay, flow cytometry and TUNEL analysis of apoptosis). The investigator was blinded to the group allocation during the experiment.

### Tube formation of HUVECs assay

Angiogenesis of HUVECs was assessed using a capillary tube forming assay. HUVECs (30,000 cells/well) were then seeded in 96 wells plates covered with growth factor reduced Matrigel (356,230; BD Biosciences, San Jose, CA, USA). After 6 h, capillary like tube formation was photographed. Tube length was quantified by Image J software (National Institutes of Health, NIH). Each experiment was performed in triplicate.

### EdU and migration assay

For proliferation evaluation, HUVECs were labeled with EdU for detection as described previously. [12] HUVECs (2 × 10^4^ cells/well) were seeded in 24 wells plates and they were incubated with EdU labeling reagent (Invitrogen, in 1:1,000 dilution) for 24 h. Then cells were fixed with 4% paraformaldehyde for 30 min at room temperature, and treated with Triton X‐100 for another 20 min, and then washed three times with PBS. Click‐iT EdU Alexa Fluor 555 Imaging Kit (Invitrogen) was used according to manufacturer's instructions. Finally, cells were stained with 4′, 6‐diamidino‐2‐phenylindole (DAPI, Sigma‐Aldrich, St. Louis, MO, USA). Images were analyzed by Image J software. All samples were observed in triplicate.

For migration evaluation, treated HUVECs were cultured in 6 wells plates at the density of 2 × 10^5^ cells/well with 1 mL test medium. After 24 h, washed with PBS three times, and then scratched using a P200 pipette tip. Twelve hours later, cell migration was observed by microscopy and analyzed by using Image J software. All samples were observed in triplicate.

### Flow cytometry analysis of apoptosis

Apoptosis was assayed by flow cytometry as described previously. [44] For apoptosis analysis, cells were harvested with trypsin and fixed with ice cold 70% ethanol. Apoptosis was assayed with Annexin V-fluorescein isothiocyanate and propidium iodide staining (3,801,660; Sony Biotechnology, San Jose, CA, USA). Data were analyzed with a CELL Quest kit (BD Biosciences). Each experiment was performed in triplicate.

### TUNEL analysis of apoptosis

Terminal deoxynucleotidyl transferase dUTP nick end labeling (TUNEL) assays were used for cell and tissue apoptosis. A TUNEL apoptosis detection kit (Roche, USA) was used to assay apoptosis. All cell nuclei were stained with DAPI. Apoptotic cells were dyed with TUNEL positive nuclei. Samples were examined with a microscope (Zeiss LSM510 META, German). The percentage of apoptotic nuclei was calculated for further analysis. Each experiment was performed in triplicate.

### MI model establishing and exosomes injection

Our animal study protocol conforms to the Guide for the Care and Use of Laboratory Animals [National Institutes of Health, (NIH) Bethesda, MD, USA] and is approved by the Institutional Animal Care and Use Committee of the Nanjing Medical University for Laboratory Animal Medicine. Sprague Dawley rats (male, 6-8 weeks) were provided from Animal Core Facility of Nanjing Medical University (Nanjing, China). Rats were anaesthetized with intraperitoneal injection of sodium pentobarbital (50 mg/kg) and then connected to a ventilator by orotracheal intubation. After fixation, left thoracotomy was performed between the 3rd and 4th intercostal space under sterile conditions, then the left anterior descending coronary artery was ligated at 1.5 mm below the level of the inferior margin of the left auricle. After MI models successful establishing, exosomes (50ug) were equally divided into 4 portions for injection at the border of the infarction area. Carprofen (10 mg/kg) was used in postoperative analgesia to ensure that the animal did not suffer from additional pain. All surgeries and subsequent analyses were blinded for intervention.

### Assessment of cardiac function

Cardiac function was evaluated with transthoracic echocardiography (Vevo 2000 high-resolution micro-imaging system) 14 days and 28 days after exosomes therapy using isoflurane inhalation (1.5%-2%). Rats underwent intraperitoneal anesthesia with sodium pentobarbital (50 mg/kg) and placed on a heated platform in a supine position. The internal diameter of the LV was measured in the short-axis view from M-mode recordings by a 30 MHz transducer. Then left ventricular ejection fraction (LVEF) and left ventricular fraction shortening (LVFS) were analyzed using the Vevo 2000 workstation software.

### Masson trichrome staining

Slides from paraffin embedded heart tissues were stained by Masson’s trichrome to detect fibrosis. Infarct size was evaluated as the average ratio of fibrosis area to the total ventricular area. Images were captured by scanning electron microscope (SU8010, Japan) and analyzed with Image J software.

### Immunofluorescence

Immunofluorescence was performed as previously described [12]. In brief, heart tissues were collected, fixed with 4% PFA, embedded in paraffin, and sectioned. For Immunofluorescence analyses, heart sections were stained with primary antibodies against CD31 (1:200, ab7388, Abcam, Cambridge, United Kingdom) and anti-Actin (1:200, A2066, Sigma-Aldrich). DAPI was used for nuclear counterstaining.

### Statistical analysis

Continuous variables and categorical variables were described by means ± SEM and percentages. For continuous variables, student *t* test (normal distribution data) or Mann–Whitney U test (abnormal distribution data) were used. Statistical differences among more than two groups were assessed by One-way ANOVA with the Bonferroni test. A value of P < 0.05 was considered statistically significant.

## Supplementary Information

**Additional file 1.** Additional figures and tables.

## References

[1] Colombo M, Raposo G, Théry C (2014). Biogenesis, secretion, and intercellular interactions of exosomes and other extracellular vesicles. Annu Rev Cell Dev Biol.

[2] Singla DK (2016). Stem cells and exosomes in cardiac repair. Curr Opin Pharmacol.

[3] Min PK, Chan SY (2015). The biology of circulating microRNAs in cardiovascular disease. Eur J Clin Invest.

[4] Taylor DD, Gercel-Taylor C (2008). MicroRNA signatures of tumor-derived exosomes as diagnostic biomarkers of ovarian cancer. Gynecol Oncol.

[5] Lamichhane TN, Sokic S, Schardt JS, Raiker RS, Lin JW, Jay SM (2015). Emerging roles for extracellular vesicles in tissue engineering and regenerative medicine. Tissue Eng B Rev.

[6] Small EM, Frost RJ, Olson EN (2010). MicroRNAs add a new dimension to cardiovascular disease. Circulation.

[7] Iyer V, Rowbotham S, Biros E, Bingley J, Golledge J (2017). A systematic review investigating the association of microRNAs with human abdominal aortic aneurysms. Atherosclerosis.

[8] Izarra A, Moscoso I, Levent E, Cañón S, Cerrada I, Díez-Juan A, Blanca V, Núñez-Gil IJ, Valiente I, Ruíz-Sauri A (2014). miR-133a enhances the protective capacity of cardiac progenitors cells after myocardial infarction. Stem Cell Rep.

[9] Chen C, Tang Y, Sun H, Lin X, Jiang B (2019). The roles of long noncoding RNAs in myocardial pathophysiology. Biosci Rep..

[10] Liu X, Li X, Zhu W, Zhang Y, Hong Y, Liang X, Fan B, Zhao H, He H, Zhang F (2020). Exosomes from mesenchymal stem cells overexpressing MIF enhance myocardial repair. J Cell Physiol..

[11] Miller EJ, Li J, Leng L, McDonald C, Atsumi T, Bucala R, Young LH (2008). Macrophage migration inhibitory factor stimulates AMP-activated protein kinase in the ischaemic heart. Nature.

[12] Zhang Y, Zhu W, He H, Fan B, Deng R, Hong Y, Liang X, Zhao H, Li X, Zhang F (2019). Macrophage migration inhibitory factor rejuvenates aged human mesenchymal stem cells and improves myocardial repair. Aging.

[13] De R, Sarkar S, Mazumder S, Debsharma S, Siddiqui AA, Saha SJ, Banerjee C, Nag S, Saha D, Pramanik S (2018). Macrophage migration inhibitory factor regulates mitochondrial dynamics and cell growth of human cancer cell lines through CD74-NF-κB signaling. J Biol Chem.

[14] Chistiakov DA, Orekhov AN, Bobryshev YV (2016). Cardiac-specific miRNA in cardiogenesis, heart function, and cardiac pathology (with focus on myocardial infarction). J Mol Cell Cardiol.

[15] Barraclough JY, Joan M, Joglekar MV, Hardikar AA, Patel S (2019). MicroRNAs as prognostic markers in acute coronary syndrome patients-a systematic review. Cells..

[16] Chen Y, Zuo J, Chen W, Yang Z, Zhang Y, Hua F, Shao L, Li J, Chen Y, Yu Y (2019). The enhanced effect and underlying mechanisms of mesenchymal stem cells with IL-33 overexpression on myocardial infarction. Stem Cell Res Therapy.

[17] Liang X, Ding Y, Lin F, Zhang Y, Zhou X, Meng Q, Lu X, Jiang G, Zhu H, Chen Y (2019). Overexpression of ERBB4 rejuvenates aged mesenchymal stem cells and enhances angiogenesis via PI3K/AKT and MAPK/ERK pathways. FASEB J.

[18] Yu B, Kim HW, Gong M, Wang J, Millard RW, Wang Y, Ashraf M, Xu M (2015). Exosomes secreted from GATA-4 overexpressing mesenchymal stem cells serve as a reservoir of anti-apoptotic microRNAs for cardioprotection. Int J Cardiol.

[19] Ma J, Zhao Y, Sun L, Sun X, Zhao X, Sun X, Qian H, Xu W, Zhu W (2017). Exosomes Derived from Akt-modified human umbilical cord mesenchymal stem cells improve cardiac regeneration and promote angiogenesis via activating platelet-derived growth factor D. Stem Cells Transl Med.

[20] Jimenez-Puerta GJ, Marchal JA, López-Ruiz E, Gálvez-Martín P (2020). Role of mesenchymal stromal cells as therapeutic agents: potential mechanisms of action and implications in their clinical use. J Clin Med..

[21] Voisin C, Cauchois G, Reppel L, Laroye C, Louarn L, Schenowitz C, Sonon P, Poras I, Wang V, Carosella E (2020). Are the immune properties of mesenchymal stem cells from wharton's jelly maintained during chondrogenic differentiation?. J Clin Med..

[22] Yu H, Cheng J, Shi W, Ren B, Zhao F, Shi Y, Yang P, Duan X, Zhang J, Fu X, et al. Bone marrow mesenchymal stem cells-derived exosomes promote tendon regeneration via facilitating the proliferation and migration of endogenous tendon stem/progenitor cells. Acta biomaterialia. 2020. 10.1016/j.actbio.2020.01.051 32027991

[23] Lin Y, Nan J, Shen J, Lv X, Chen X, Lu X, Zhang C, Xiang P, Wang Z, Li Z (2020). Canagliflozin impairs blood reperfusion of ischaemic lower limb partially by inhibiting the retention and paracrine function of bone marrow derived mesenchymal stem cells. EBioMedicine.

[24] Huang P, Wang L, Li Q, Xu J, Xu J, Xiong Y, Chen G, Qian H, Jin C, Yu Y (2019). Combinatorial treatment of acute myocardial infarction using stem cells and their derived exosomes resulted in improved heart performance. Stem Cell Res Ther.

[25] Huang P, Wang L, Li Q, Tian X, Xu J, Xu J, Xiong Y, Chen G, Qian H, Jin C (2020). Atorvastatin enhances the therapeutic efficacy of mesenchymal stem cells-derived exosomes in acute myocardial infarction via up-regulating long non-coding RNA H19. Cardiovasc Res.

[26] Jafarinia M, Alsahebfosoul F, Salehi H, Eskandari N (2020). Ganjalikhani-Hakemi M.

[27] Haider KH, Aramini B (2020). Mircrining the injured heart with stem cell-derived exosomes: an emerging strategy of cell-free therapy. Stem Cell Res Ther.

[28] Adler-Moore J, Proffitt RT (2002). Am Bisome: liposomal formulation, structure, mechanism of action and pre-clinical experience. J Antimicrob Chemother..

[29] Zhang M, Liang J, Yang Y, Liang H, Jia H, Li D (2020). Current trends of targeted drug delivery for oral cancer therapy. Front Bioeng Biotechnol.

[30] Park DJ, Yun WS, Kim WC, Park JE, Lee SH, Ha S, Choi JS, Key J, Seo YJ (2020). Improvement of stem cell-derived exosome release efficiency by surface-modified nanoparticles. J Nanobiotechnol.

[31] Zhang Y, Bi J, Huang J, Tang Y, Du S, Li P (2020). Exosome: a review of its classification, isolation techniques, storage, diagnostic and targeted therapy applications. Int J Nanomed.

[32] Rong S, Wang L, Peng Z, Liao Y, Li D, Yang X, Nuessler AK, Liu L, Bao W, Yang W (2020). The mechanisms and treatments for sarcopenia: could exosomes be a perspective research strategy in the future?. J Cachexia Sarcopenia Muscle..

[33] Kalluri R, LeBleu VS. The biology function and biomedical applications of exosomes. Science (New York, NY). 2020; 367. 10.1126/science.aau6977 PMC7717626 32029601

[34] Ullah M, Ng NN, Concepcion W, Thakor AS (2020). Emerging role of stem cell-derived extracellular microRNAs in age-associated human diseases and in different therapies of longevity. Ageing Res Rev.

[35] Pinet F, Bauters C, Bär C, Thum T (2019). Letter by Pinet et al Regarding Article, "Comparative analysis of circulating noncoding RNAs versus protein biomarkers in the detection of myocardial injury". Circ Res.

[36] Li M, Ding W, Tariq MA, Chang W, Zhang X, Xu W, Hou L, Wang Y, Wang J (2018). ncx1A circular transcript of gene mediates ischemic myocardial injury by targeting miR-133a-3p. Theranostics.

[37] Wüst S, Dröse S, Heidler J, Wittig I, Klockner I, Franko A, Bonke E, Günther S, Gärtner U, Boettger T (2018). Metabolic maturation during muscle stem cell differentiation is achieved by miR-1/133a-mediated inhibition of the Dlk1-Dio3 mega gene cluster. Cell Metab.

[38] Wang SS, Wu LJ, Li JJ, Xiao HB, He Y, Yan YX (2018). A meta-analysis of dysregulated miRNAs in coronary heart disease. Life Sci.

[39] Wang F, Long G, Zhao C, Li H, Chaugai S, Wang Y, Chen C, Wang DW (2013). Plasma microRNA-133a is a new marker for both acute myocardial infarction and underlying coronary artery stenosis. J Transl Med.

[40] Hausenloy DJ, Yellon DM (2013). Myocardial ischemia-reperfusion injury: a neglected therapeutic target. J Clin Investig.

[41] Hausenloy DJ, Yellon DM (2007). Reperfusion injury salvage kinase signalling: taking a RISK for cardioprotection. Heart Fail Rev.

[42] Li N, Zhou H, Tang Q (2018). miR-133: a suppressor of cardiac remodeling?. Front Pharmacol.

[43] Sang HQ, Jiang ZM, Zhao QP, Xin F (2015). MicroRNA-133a improves the cardiac function and fibrosis through inhibiting Akt in heart failure rats. Biomed Pharmacother..

[44] Sun R, Xiang T, Tang J, Peng W, Luo J, Li L, Qiu Z, Tan Y, Ye L, Zhang M (2020). 19q13 KRAB zinc-finger protein ZNF471 activates MAPK10/JNK3 signaling but is frequently silenced by promoter CpG methylation in esophageal cancer. Theranostics.

