# Supplementary material for: Macrophage migration inhibitory factor facilitates the therapeutic efficacy of mesenchymal stem cells derived exosomes in acute myocardial infarction through upregulating miR-133a-3p
Source: J Nanobiotechnology. 2021 Feb 27;19:61. doi: 10.1186/s12951-021-00808-5 (PMC7916292; doi:10.1186/s12951-021-00808-5)

Supplementary table 1. Primer list

Primers	Sequences (5'–3')
hsa-miR-133a-3p Forward	ACACTCCAGCTGGGTTTGTCCCCTTCAAC
hsa-miR-133a-3p Reverse	TGGTGTCGTGGAGTCG
U6 Forward	CTCGCTTCGGCAGCACA
U6 Reverse	AACGCTTCACGAATTTGCGT
Cel-miR-39-3p Forward	GGGTCACCGGGTGTAAATC
Cel-miR-39-3p Reverse	GAGAGGAGAGGAAGAGGGAA

Supplementary table 2. Sequences list of miR-133a-3p mimics or inhibitor

	Sequences (5'–3')
hsa miR-133a-3p mimics	UUUGGUCCCCUUCAACCAGC
hsa miR-133a-3p mimics NC	UUUUCCGAACGUUCACGUTT
hsa miR-133a-3p inhibitor	CCAGTACATCGCGGTGCACGT
hsa miR-133a-3p inhibitor NC	CAGUACUUUUGUGUAGUACAA

Supplementary table 3. Sequences list of MIF and siMIF

MIF and siMIF	Sequences (5'–3')
MIF	ATGCCGATGTTCATCGTAAACACCAACG TGCCCCGCGCCTCCGTGCCGGACGGGTT CCTCTCCGAGCTCACCCAGCAGCTGGC GCAGGCCACCGGCAAGCCCCCCCAGTA CATCGCGGTGCACGTGGTCCCGGACCA GCTCATGGCCTTCGGCGGCTCCAGCGAG CCGTGCGCGCTCTGCAGCCTGCACAGCA TCGGCAAGATCGGCGGCGCGCAGAACC GCTCCTACAGCAAGCTGCTGTGCGGCCT GCTGGCCGAGCGCCTGCGCATCAGCCC GGACAGGGTCTACATCAACTATTACGAC ATGAACGCGGCCAATGTGGGCTGGAAC AACTCCACCTTCGCC
siMIF	AGTACATCGCGGTGCACGT

Supplementary Figure 1. HUVECs and H9C2s were transfected by transfection reagents. (A) qRT-PCR showed that miR-133 mimics or inhibitor transfected HUVECs sucessfully. **(B)** qRT-PCR showed that miR-133 mimics or inhibitor transfected H9c2 cells sucessfully. (n=3). All data are mean ± SEM. Statistical analysis was performed with t test. *P < 0.05, **P < 0.01, ****P < 0.0001.

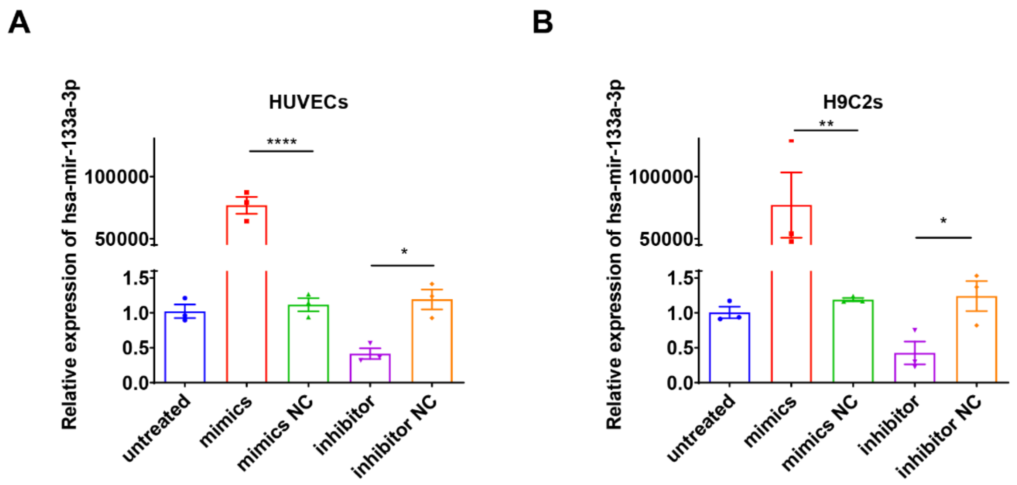

Supplementary Figure 2. Gain and loss function of exosomal miR-133a-3p on pro-angiogenisis, proliferation, and apoptosis in HUVECs and H9c2 cells. (A) Quantification analysis angiogenic effects in HUVECs with incubation of MIF-Exo and miR-133a-3p inhibitor (n = 3 biological replicates for each group). **(B)** Quantification analysis of proliferation effects in HUVECs with incubation of MIF-Exo and miR-133a-3p inhibitor (n = 3 biological replicates for each group; 5 random fields for each biological replicate). **(C)** Quantification analysis Apoptotic ability in H9c2 cells with incubation of MIF-Exo and miR-133a-3p inhibitor (n = 3 biological replicates for each group). Continuous variables were described by means ± SEM and percentages. **P*<0.05; *****P* < 0.0001; NS, not significant.

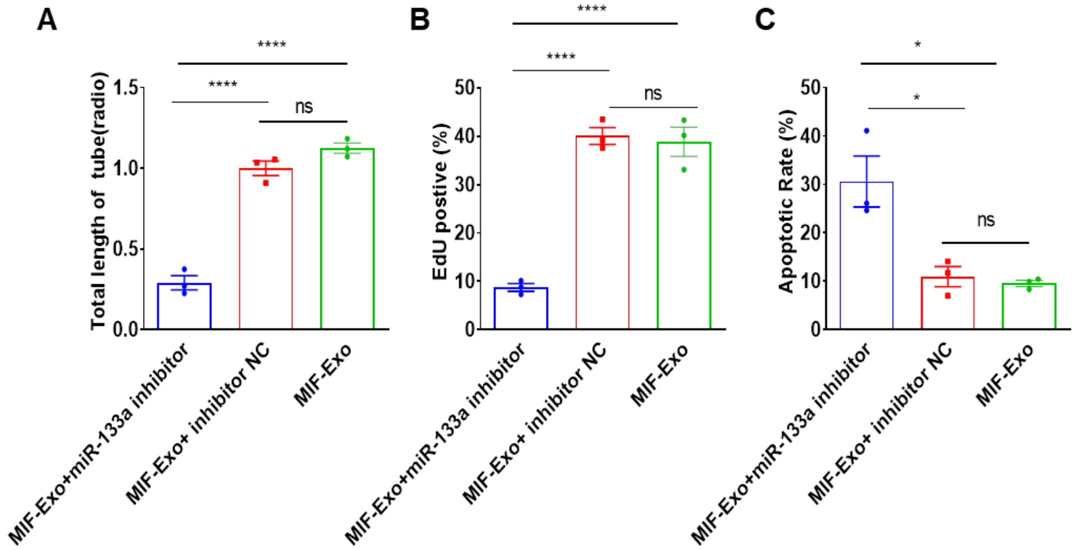

Supplement: Supplementary file 1 — Additional file 1. Additional figures and tables. [file 12951_2021_808_MOESM1_ESM.pdf]
